# The role of psychological strengths in the relationship between self- and external- regulation of behavior and psychological well-being of university students

**DOI:** 10.3389/fpsyg.2026.1641359

**Published:** 2026-04-02

**Authors:** Elkin O. Luis Garcia, Leyre San Martín-Iñiguez, José Manuel Martínez-Vicente, Jesús de la Fuente

**Affiliations:** 1 Department of Psychology, School of Education and Psychology, University of Navarra, Pamplona, Spain; 2 Department of Psychology, School of Psychology, University of Almería, Almería, Spain

**Keywords:** regulation, nonregulation, dysregulation, psychological strengths, psychological well-being, self- vs. external-regulation theory

## Abstract

**Background:**

Previous research has identified associations among regulatory processes, psychological strengths, and well-being. However, empirical evidence regarding the predictive and mediating mechanisms underlying these relationships is limited. The present study aimed to (1) examine the predictive relationships between self-external regulation of behavior and psychological strengths and (2) analyze the mediating role of psychological strengths in the relationship between self-external regulation and psychological well-being.

**Methods:**

A sample of 453 university students completed validated self-report instruments. An ex post facto, cross-sectional design was used to conduct correlational analyses, hierarchical regression models, and structural equation modeling to test the proposed hypotheses.

**Results:**

Higher levels of self- and external regulation significantly and positively predicted psychological strengths, both globally and across specific dimensions. Nonregulation and dysregulation, on the other hand, showed negative associations. In turn, psychological strengths significantly predicted psychological well-being and accounted for a substantial proportion of its variance. Structural equation modeling confirmed the mediating role of psychological strengths in the relationship between regulatory factors and well-being. Regulation demonstrated stronger predictive weight than nonregulatory and dysregulatory factors.

**Conclusion:**

These findings provide empirical support for the Self- vs. External-Regulation Behavior Theory by clarifying its predictive and mediational mechanisms. The results underscore the importance of considering personal and contextual regulatory processes when examining psychological strengths and well-being. They also suggest implications for preventive, educational, clinical, and health-related interventions.

## 1 Introduction

### 1.1 Psychological well-being

Recent research has increasingly emphasized the multifaceted nature of psychological well-being. Since the emergence of Positive Psychology, scholarly attention has focused on understanding human efforts, achievements, and potential (Wood et al., 2011). At this point, well-being has traditionally been conceptualized through two complementary models: the hedonic and the eudaimonic.

The hedonic model emphasizes the pursuit of pleasure and the avoidance of discomfort. It typically comprises three components—positive affect, negative affect, and life satisfaction (Diener et al., 1985)—which together capture individuals’ subjective evaluation of their hedonic well-being (Disabato et al., 2016). On the other hand, the eudaimonic model focuses on the development of personal potentials and virtues (Ryff et al., 1995). Ryff and Singer (1996) proposed a model that conceptualizes eudaimonic well-being in terms of dimensions such as autonomy, environmental mastery, personal growth, purpose in life, and self-acceptance.

Although conceptually distinct, recent studies highlight the value of integrating these two perspectives, as hedonic and eudaimonic factors appear to function as related subcomponents within a broader overarching construct of well-being (Disabato et al., 2016). This integrative view has motivated research into the psychological variables that shape both forms of well-being. For example, studies have demonstrated that self-control is positively linked to hedonic and eudaimonic well-being (Li et al., 2022). A plausible explanation is that self-control facilitates the pursuit of long-term, meaningful goals (supporting eudaimonia), while also helping individuals manage impulsive behaviors that may undermine positive emotions (supporting hedonia). Additionally, eudaimonic motives show positive associations with self-control, whereas hedonic motives tend to relate negatively to it (Zeng and Chen, 2020), suggesting that these motivational orientations may differentially influence regulatory processes.

A growing body of evidence has also highlighted the influence of personal values on well-being, particularly through processes of self-affirmation. Congruence between individual values and contextual values fosters higher well-being (Sagiv and Schwartz, 2021). Positive associations have additionally been observed between psychological well-being and both academic performance (Bücker et al., 2018; Hossain et al., 2022) and occupational functioning (Di Fabio, 2017; Tims et al., 2013). These findings suggest that psychological well-being may operate as a facilitator of optimal functioning across life domains and contexts.

Conversely, research on young populations has shown consistent negative relationships between well-being and health risk behaviors such as substance use, unhealthy diet, sedentary lifestyle, sleep deprivation, and depressive symptomatology (Calderon et al., 2021). Well-being has also been found to buffer the psychological factors involved in physical illness and to promote more complete recovery processes (DuPont et al., 2020).

Despite the considerable knowledge accumulated, important gaps remain. Specifically, the psychological and contextual mechanisms that modulate, mediate, or predict psychological well-being are still not fully understood. Identifying these mechanisms—particularly among young populations—may contribute to the design of interventions aimed at promoting optimal psychological functioning.

### 1.2 Psychological strengths

Psychological strengths, or character strengths, are relatively stable personal resources that enable individuals to perform at their best (Gander et al., 2020; Liu et al., 2022). Seligman (2002) distinguishes three life orientations conducive to happiness: the pleasant life, the engaged life, and the meaningful life. In this context, Peterson and Seligman (2004) developed the most widely used scientific classification of character strengths, identifying 24 strengths grouped into six core virtues: justice, humanity, wisdom and knowledge, transcendence, temperance, and courage. This framework, known as the Values in Action (VIA) classification, was designed to represent universal positive qualities identifiable across cultures and historical periods (Dahlsgaard et al., 2005).

Recent research stresses the importance of examining how both individual and contextual characteristics shape the development of character strengths. Furnham and Lester (2012), for example, reported that personality factors—particularly extraversion—are strong predictors of strengths and virtues. They also observed gender differences, with young women tending to show higher development in emotional and interpersonal strengths, whereas men scored higher on cognitive strengths. Similarly, Cosentino and Castro Solano (2012) conducted a cultural context study of character strengths and found that these strengths varied according to ethnicity group.

### 1.3 Strengths and psychological well-being

A substantial body of research has demonstrated a robust positive relationship between character strengths and well-being (DuPont et al., 2020; Dahlsgaard et al., 2005). Zhang and Chen (2018) additionally identified that the actual use of strengths mediates the relationship between character strengths and well-being, suggesting that strengths exert their positive effects through behavioral expression.

Evidence from child and adolescent populations further indicates favorable associations between strengths and indicators of well-being across diverse cultural contexts (Khanna et al., 2021), along with negative associations between strengths and behavioral problems or risk factors (Jabbari et al., 2021). Among university students, strengths have been linked not only to higher well-being (Green, 2022; Zhang and Chen, 2018) but also to stable academic performance over time (Gander et al., 2020). Consequently, interventions based on psychological strengths have repeatedly shown increases in both subjective and psychological well-being (Green, 2022; Hausler et al., 2017; Khanna et al., 2021).

Taken together, existing evidence indicates that psychological strengths are closely tied to positive functioning and higher levels of mental health (Azañedo et al., 2021; Zhang and Chen, 2018). These strengths are therefore relevant for understanding the psychological (e.g., meaning, personal growth) and emotional (e.g., positive affect) dimensions of well-being (Liu et al., 2022).

### 1.4 Regulatory behavior

Self-regulation refers to the capacity to plan, monitor, and evaluate actions directed toward achieving desired outcomes (de la Fuente et al., 2022a; Inzlicht et al., 2021). Several theoretical models have contributed to the conceptualization of behavioral regulation. For example, the Self-Regulation Model (Carver and Scheier, 2001) draws on cybernetic principles and distinguishes four components: goal setting, monitoring of current behavior, detection of threats or discrepancies, and mechanisms to reduce gaps between actual and desired states (Inzlicht et al., 2021). The Goal Systems Theory (Kruglanski et al., 2002) examines how the structure and organization of goals influence self-regulatory processes.

The Social Cognitive Theory (Bandura, 1986) emphasizes the interplay of personal, behavioral, and environmental factors in human functioning, proposing that individuals act to maintain a sense of agency. Derived from this theory, models such as Self-Determination Theory (Deci and Ryan, 1985a, 1985b, 2000) describe a progression from externally regulated to self-regulated behavior (de la Fuente et al., 2022a). Likewise, the Theory of Self-Regulated Learning (Zimmerman and Schunk, 2001, 2011) delineates three phases—forethought, performance, and self-reflection—through which individuals become aware of and regulate their learning processes (de la Fuente et al., 2022a).

Although each of these models has contributed to advancing the conceptual understanding of self-regulation, they share certain limitations. Specifically, they tend to (1) underestimate the influence of external regulation on the individual’s regulatory behavior, (2) fail to distinguish between different types of regulatory behavior (regulatory, non-regulatory, and dysregulatory), and (3) overlook self-regulation as a predictive variable connecting macro-level processes (de la Fuente et al., 2022a).

### 1.5 Model of self- vs. external regulation behavior

In response to these limitations, the Self- vs. External-Regulation of Behavior Theory (de la Fuente et al., 2021, 2022a, 2022b) proposes a more integrative framework. This model explains behavior based on the interplay between individuals’ own regulatory characteristics and the regulatory qualities of their context (de la Fuente et al., 2022b; de la Fuente and Kauffman, 2025).

Building upon previous explanatory models—including the Motivated Strategies for Learning Questionnaire (Pintrich and De Groot, 1990), the Self-Regulated Learning Model (Zimmerman, 1989), Self-Determination Theory (Deci and Ryan, 1987), and Miller and Brown’s Behavioral Self-Regulation Model (Miller and Brown, 1991)—this theory seeks to clarify how self-regulation and external contextual factors jointly shape behavioral regulatory tendencies across educational, clinical, health, social, and organizational settings (de la Fuente et al., 2022a, 2022b).

The model proposes three levels of self-regulatory behavior: Self-Regulation (SRG), Non-Regulation (NRG), and Dys-Regulation (DRG) (de la Fuente et al., 2019; de la Fuente-Arias, 2017). SRG reflects effective behavioral regulation; NRG reflects a lack of proactivity (neutral state); and DRG reflects inadequate behavioral management, characterized by negative proactivity and difficulty controlling thoughts, emotions, and actions.

Similarly, external regulation is conceptualized in three levels: External Regulation (ERG), External Non-Regulation (ENRG), and External Dys-Regulation (EDRG). ERG provides signals and supports that facilitate self-regulated behavior; ENRG lacks cues that promote either regulation or dysregulation; and EDRG involves contextual conditions that encourage negative or maladaptive behavioral tendencies.

The interaction between personal and contextual regulation is assumed to shape individuals’ overall regulatory tendencies, ranging from highly dysregulated to highly regulated states (de la Fuente et al., 2022a, 2022b). However, the predictive and combined effects of these regulatory levels on psychological strengths and psychological well-being remain insufficiently explored. Although earlier studies have identified positive associations between strengths and well-being (e.g., Dahlsgaard et al., 2005; Furnham and Lester, 2012; de la Fuente et al., 2021), the joint role of self-regulation and external regulation in these relationships has not yet been fully examined.

### 1.6 Purpose

Although previous research has indicated links among regulation processes, psychological strengths, and well-being, empirical evidence on the predictive and mediating mechanisms underlying these associations remains limited. Therefore, the present study aims (1) to examine the predictive relationships between self–external regulation of behavior and psychological strengths, and (2) analyze the mediating role of psychological strengths in the relationship between self–external regulation and psychological well-being.

The study hypothesizes:

1. that higher levels of self- and external regulation will positively predict psychological strengths, both globally and across specific dimensions, and
2. that psychological strengths will mediate the relationship between self–external regulation and psychological well-being, enhancing the positive impact of regulation on well-being outcomes.

## 2 Methods

A prospective and transversal ex post facto design was used (Ato et al., 2013). This design can help overcome situations in which the variable of interest has already occurred and/or it would be unethical to cause it. The advantages of this method are clear, as it ensures that the independent variable precedes the dependent variable in the analysis. The present study was conducted as a cross-sectional investigation due to the impracticality of longitudinal follow-up and data collection over a brief timeframe.

### 2.1 Participants

Four hundred and fifty-three undergraduates currently doing a degree at public universities in Spain participated in the study. The sample was selected using convenience sampling and comprised students from programs in Psychology, Primary Teaching, and Educational Psychology. Most participants were women (71.4%) and their ages ranged from 18 to 25 years (mean age 20.66; *σ* = 1.86).

### 2.2 Instruments

#### 2.2.1 Self- vs. external regulation of behavior in health (SRH-ERH)

The SRH-ERH Questionnaire (de la Fuente et al., 2022b) was used to measure the regulation of health-related behaviors in individual and contextual domains. The questionnaire consists of 3 subscales with 6 items each to assess regulatory, nonregulatory, and dysregulatory aspects of self- regulation, and another set of 3 subscales with 6 items each to assess regulatory, nonregulatory, and dysregulatory aspects of external regulation. The factor structure of the questionnaire in this sample was consistent Chi-square = 1647.619, *p* < 0.001; df(702–118) = 584; CH/DF = 2,821; CFI = 0.958; GFI = 0.938; IFI = 0.926; TLI = 0.928; CFI = 0.926, RMSEA = 0.023; RSMR = 0.052; Hoelter = 1,294 (*p* < 0.05), 1,345 (*p* < 0.01). The total reliability value was also acceptable (total alpha = 0.897; omega = 0.868), as were the subscale consistency values: Self-Regulation in Health (SRH) = 0.901; Nonregulation in Health (NRH) = 0.785; Dysregulation in Health (DRH) = 0.873; External Regulation in Health (ERH) = 0.950; External Non-Regulation in Health (ENH) = 0.805; and External Dysregulation in Health (EDH) = 0.939. The present study reassessed internal consistency, yielding a satisfactory total Cronbach’s alpha of 0.88. This result supports the reliability of the instrument in this sample.

#### 2.2.2 The VIA inventory of strengths

The abbreviated version of the VIA Inventory of Strengths (VIA-IS-72) (Peterson and Seligman, 2004) comprises 72 items, 24 factors, and 6 dimensions (wisdom and knowledge, courage, humanity, justice, temperance and transcendence), that allow subjects to self-asses their character strengths. The internal consistency of the 24 VIA-72 subscales, as measured by Cronbach’s alpha, ranged from 0.60 (Leadership) to 0.87 (Humor and Perseverance), with an average alpha of 0.75 across subscales. “Three Likert-style items with options ranging from 1 (“Very Much Unlike Me”) to 5 (“Very Much Like Me”) evaluate each character strength. According to assessments of internal consistency, reliability, and validity, the VIA-72 is essentially equivalent to the original, long version of the VIA-IS, as confirmed by its developers (Peterson and Seligman, 2004). The Spanish version of VIA-72 employed for this study demonstrated comparable psychometric values to the ones presented in the original version in English. In this study, the second-order model showed good fit (χ^2^ = 35.0, *p* = 0.07, df = 24; χ^2^/df = 1.46; CFI = 0.96; TLI = 0.94; RMSEA = 0.05; SRMR = 0.06). The reliability coefficients were: Wisdom α = 0.79, Courage α = 0.87, Humanity α = 0.47, Justice α = 0.86, Temperance α = 0.60, Transcendence α = 0.82, Total Strengths α = 0.97.

#### 2.2.3 Psychological well-being

The Spanish version of the Psychological Well-Being Scale (Díaz et al., 2006) was employed to assess the participants’ psychological well-being. This instrument comprises 29 items designed to evaluate 6 dimensions (Ryff, 1989a, 1989b). The scale exhibits a consistent confirmatory factor structure (χ^2^ = 845,593, df = 113, χ^2^/df = 7.48, *p* < 0.001, RMR = 0.029, NFI = 0.937, RFI = 0.942, IFI = 0.961, TLI = 0.956, CFI = 0.964, RMSEA = 0.05). The reliability coefficients are appropriate (alpha total = 0.905; omega = 0.886) and the internal consistency of each dimension was determined as follows: self-acceptance (α = 0.82), autonomy (α = 0.80), positive relations (α = 0.79), personal growth (α = 0.74), environmental mastery (α = 0.70), and purpose in life (α = 0.85). The present sample was reassessed for internal consistency, yielding a total Cronbach’s alpha of 0.91, which supports the reliability of the measure. Participants rated their responses by means of a 5-item Likert scale, where higher scores indicated higher levels of psychological well-being in each dimension.

### 2.3 Procedure

Once the corresponding informed consent obtained through an online platform (de la Fuente, 2015), university students volunteered to complete the validated questionnaires. The teaching-learning processes of the five subjects in which the participants were enrolled in 2016 and 2017 were evaluated. Data were collected from September 2018 to June 2020. This entire procedure was approved by the Ethics Committee of the University of Navarra under reference 2018.170.

### 2.4 Data analysis

Three types of analyses were conducted (preliminary analysis, predictive analysis and structural equation model) with the standard assumptions for regression analyses assessed beforehand.

#### 2.4.1 Preliminary analysis

First, data quality was examined by screening for missing values and outliers. To ensure the robustness and interpretability of the statistical results, cases with missing data were removed prior to conducting the main analyses. IBM SPSS Statistics version 26 (IBM Corp., 2019) was used to assess the pattern and magnitude of missing data and to determine whether they occurred systematically or at random.

Univariate outliers were identified by calculating standardized scores (z-scores) for each variable, excluding values exceeding ±3 standard deviations, in accordance with Tabachnick and Fidell (2007). Additionally, the Mahalanobis distance (D^2^) was computed to detect atypical multivariate cases by estimating each individual’s multidimensional distance from the centroid of the observed variables (Lohr and Velasco, 2020). This procedure enabled the identification of significant deviations from the typical multivariate profile of the dataset. Previous research has suggested either removing univariate and multivariate outliers or reassigning them to the nearest extreme value (Weston and Gore, 2006).

Normality assumptions were assessed through the examination of skewness and kurtosis indices for each observed variable. The results indicated deviations from the univariate normality criteria. Additionally, while some residuals departed from normality, the large sample size (*N* = 453) ensures the robustness of ordinary least squares (OLS) estimates under the Central Limit Theorem. Therefore, these deviations were not considered sufficient to compromise the validity of subsequent inferential analyses.

Multivariate normality was further examined using Mardia’s multivariate kurtosis index (Mardia, 1970). In addition, collinearity diagnostics revealed no concerns regarding multicollinearity, as no pair of predictors simultaneously exhibited high variance proportions (>0.50) within the same dimension. Overall, the assumptions regarding linearity, independence of errors, multicollinearity, recursion, and interval-level measurement were satisfactorily met. Regarding sample size, the present study met the criteria suggested by Kline (2005) as the final sample comprised 453 participants. Consequently, the use of linear regression was deemed statistically appropriate for the subsequent analyses.

#### 2.4.2 Associative and predictive analyses

To test Hypothesis 1, Spearman rank-order correlations were computed to examine associative relationships, given the absence of univariate normality. Subsequently, multiple linear regression analyses were conducted to assess predictive relationships between the study variables.

#### 2.4.3 Mediation analysis

Mediation models were tested using AMOS (v.22) (Ho, 2006). First, the model fit was assessed through the examination of the relationship between chi-square and the degrees of freedom, and then through the Comparative Fit Index (CFI), the Normed Fit Index (NFI), the Incremental Fit Index (IFI), the Relative Fit Index (RFI), and the Tucker–Lewis Index (TLI).

Next, the findings from the original scale were replicated. Furthermore, the Hoelter index was employed to determine whether a sufficient number of participants was included in the sample. The present study employed the beta coefficients proposed by Keith (2006) as research benchmarks for direct effects: values less than 0.05 were considered to be too small to be meaningful, values between 0.05 and 0.10 were considered to be small but meaningful, values between 0.10 and 0.25 were considered moderate, and values above 0.25 were considered large. For indirect effects, Kelley and Preacher’s (2012) definition was used, and 0.003, 0.01, and 0.06 were established as the threshold values for small, moderate and large indirect effects, respectively.

## 3 Results

### 3.1 Previous analysis: descriptive

Descriptive statistics showed values that suggested a normalized distribution of the variables analyzed in the study sample (Supplementary Table S1). The results show the overall average of each of the six states of Self-External Regulation under study, where the highest mean score were observed for Self-Regulation (*M*SRG = 4.01, SD = 0.31) and External Regulation (*M*ERG = 3.81, SD = 0.41), while the lowest values were found for External Nonregulation (*M*ENRG = 2.43, SD = 0.45) and External Dysregulation (*M*EDRG = 2.47, SD = 0.45).

The psychological strengths presented a similar case, where the dimensions with the highest average in the sample were related to justice (*M* = 4.00, SD = 0.27), humanity (*M* = 3.96, SD = 0.27), and courage (*M* = 3.81, SD = 0.26), while those with the lowest values were wisdom and knowledge (*M* = 3.65, SD = 0.28), temperance (3.56, SD = 0.27), and transcendence (*M* = 3.51, SD = 0.28). The factors with the highest averages were integrity (*M* = 4.25; SD = 0.28), kindness (*M* = 4.18, SD = 0.27), citizenship (*M* = 4.11; SD = 0.30), and leadership (*M* = 3.99; SD = 0.30), while those with the lowest averages were self-regulation (*M* = 3.42; SD = 0.35), forgiveness and mercy (*M* = 3.40, SD = 0.43), love of learning (*M* = 3.32, SD = 0.32), and spirituality (*M* = 2.77; SD = 0.48).

With respect to psychological well-being, the highest averages among the study participants were related to personal growth (*M* = 4.89; SD = 0.39), positive relationships with others (*M* = 4.54; SD = 0.46), purpose in life (*M* = 4.51; SD = 0.46) and self-acceptance (*M* = 4.54; SD = 0.46), while the lowest values were in the domains of environmental mastery (*M* = 4.17; SD = 0.41) and autonomy (*M* = 4.03, SD = 0.41).

### 3.2 Linear association relationships

#### 3.2.1 Self-external regulation of behavior and psychological strengths

Overall, the bivariate associations revealed that the strongest and most significant associations were between Self-Regulation (SRG) and External Regulation (ERG) factors with all the dimensions of psychological strengths. This highlights the importance of both individual and contextual regulatory characteristics, which are associated with strengths, and the fact that nonregulatory contexts are negatively associated with psychological strengths. The present study revealed how some specific associations require special attention. First, the strength of justice was negatively associated with Self Nonregulation (*r* = −0.10, *p* < 0.03), External Nonregulation (*r* = −0.16, *p* < 0.001), Self Dysregulation (*r* = −0.10, *p* < 0.05) and External Dysregulation (*r* = −0.11, *p* < 0.05). Furthermore, the strength of transcendence was positively associated with Self Dysregulated Behavior (*r* = 0.16, *p* < 0.00) and External Dysregulation (*r* = 0.18, *p* < 0.00) (Table 1).

**Table 1:** Association relationships between self-external regulation and psychological strengths dimensions (*n* = 453).

Variable	SRG	NRG	DRG	ERG	ENRG	EDRG
D1. Wisdom and Knowledge	0.50 (<0.001)**	−0.08 (0.10)	0.04 (0.44)	0.31 (<0.001)**	−0.10 (0.04)*	−0.04 (0.40)
D2. Courage	0.53 (<0.001)**	−0.06 (0.18)	0.06 (0.22)	0.39 (<0.001)**	−0.14 (0.002)**	−0.02 (0.69)
D3. Humanity	0.47 (<0.001)**	−0.10 (0.03)*	−0.02 (0.65)	0.43 (<0.001)**	−0.22 (<0.001)**	−0.05 (0.29)
D4. Justice	0.46 (<0.001)**	−0.14 (0.004)**	−0.10 (0.03)*	0.38 (< 0.001)**	−0.20 (<0.001)**	−0.11 (0.02)*
D5. Temperance	0.39 (<0.001)**	−0.09 (0.05)	−0.06 (0.17)	0.34 (<0.001)**	−0.09 (0.05)	−0.04 (0.43)
D6. Trascendence	0.49 (<0.001)**	−0.02 (0.69)	0.13 (0.006)**	0.36 (<0.001)**	−0.10 (0.03)*	0.02 (0.70)

**The correlation is significant at the 0.01 level (two-tailed).

*The correlation is significant at the 0.05 level (two-tailed).

SRG, Self-Regulation; NRG, Nonregulation; DGR, Dysregulation; ER, External Regulation; ENRG, External Nonregulation; EDRG, External Dysregulation Regulation.

The bold values indicate the statistically significant results in the analyses.

Additionally, the associations between Self and External Regulation and the constituent factors of psychological strengths were, in general, significant and positive. Significant negative associations were evident between Self and External Dysregulation and the integrity factor, whereas there were positive associations with hope and spirituality. The findings indicate a negative correlation between Self-Dysregulation and citizenship, whereas a positive correlation is observed with humor and Self-Regulation (as a temperance dimension). Additionally, both Self and External Nonregulation showed a negative correlation with the factors of integrity and prudence. Moreover, a negative relationship is identified between Self-Nonregulation and citizenship. Concerning External Nonregulation, adverse associations are observed with vitality, love, kindness, fairness, and leadership (Supplementary Table S2).

#### 3.2.2 Self-external regulation of behavior and well-being

The correlations between types of Self-External Regulation and factors related to psychological well- being showed a clear and consistent trend toward a significant positive association in all factors between Self-External Regulation dimensions and psychological well-being. Additionally, Nonregulation and Dysregulation (self and external) appeared to be significantly and negatively associated with well-being factors. Purpose in life had the strongest association with both Self and External Regulation, while autonomy was the well-being factor that obtained the weakest association (Table 2).

**Table 2:** Correlations between self and external regulation types and factors of psychological well-being factors (*n* = 453).

Variable	SRG	NRG	DRG	ERG	ENRG	EDRG
F1. Self-acceptance	0.48 (<0.001)**	−0.15 (0.001)**	0.01 (0.85)	0.40 (<0.001)**	−0.24 (<0.001)**	−0.02 (0.71)
F2. Positive relations with others	0.25 (<0.001)**	−0.18 (<0.001)**	−0.19 (<0.001)**	0.34 (<0.001)**	−0.34 (<0.001)**	−0.19 (<0.001)**
F3. Autonomy	0.16 (0.001)**	−0.18 (<0.001)**	−0.11 (0.02)*	0.12 (0.01)*	−0.20 (<0.001)**	−0.12 (0.01)*
F4. Environmental mastery	0.39 (<0.001)**	−0.14 (0.002)**	−0.07 (0.12)	0.37 (<0.001)**	−0.27 (<0.001)**	−0.07 (0.16)
F5. Personal growth	0.36 (<0.001)**	−0.30 (<0.001)**	−0.20 (<0.001)**	0.28 (<0.001)**	−0.30 (<0.001)**	−0.17 (<0.001)**
F6. Purpose in life	0.56 (<0.001)**	−0.14 (0.003)**	−0.01 (0.91)	0.43 (<0.001)**	−0.24 (<0.001)**	−0.03 (0.50)

**The correlation is significant at the 0.01 level (two-tailed).

*The correlation is significant at the 0.05 level (two-tailed).

SRG, Self-Regulation; NRG, Nonregulation; DGR, Dysregulation; ER, External Regulation; ENRG, External Nonregulation; EDRG, External Dysregulation Regulation.

The bold values indicate the statistically significant results in the analyses.

#### 3.2.3 Psychological strengths and well-being

The analysis revealed significant positive relationships (Table 3) between the dimensions of psychological strength and the total score for the factors related to psychological well-being.

**Table 3:** Associations between dimensions of strengths and factors of psychological well-being (*n* = 453).

Variable	D1. Wisdom and knowledge	D2. Courage	D3. Humanity	D4. Justice	D5. Temperance	D6. Trascendence
F1. Self-acceptance	0.45 (<0.001)**	0.56 (<0.001)**	0.59 (<0.001)**	0.41 (<0.001)**	0.37 (<0.001)**	0.56 (<0.001)**
F2. Positive relations with others	0.22 (<0.001)**	0.31 (<0.001)**	0.45 (<0.001)**	0.32 (<0.001)**	0.25 (<0.001)**	0.26 (<0.001)**
F3. Autonomy	0.27 (<0.001)**	0.32 (<0.001)**	0.29 (<0.001)**	0.25 (<0.001)**	0.16 (0.001)**	0.18 (<0.001)**
F4. Environmental mastery	0.38 (<0.001)**	0.46 (<0.001)**	0.52 (<0.001)**	0.35 (<0.001)**	0.36 (<0.001)**	0.47 (<0.001)**
F5. Personal growth	0.39 (<0.001)**	0.43 (<0.001)**	0.45 (<0.001)**	0.39 (<0.001)**	0.28 (<0.001)**	0.33 (<0.001)**
F6. Purpose in life	0.51 (<0.001)**	0.61 (<0.001)**	0.57 (<0.001)**	0.44 (<0.001)**	0.42 (<0.001)**	0.57 (<0.001)**

**The correlation is significant at the 0.01 level (two-tailed).

*The correlation is significant at the 0.05 level (two-tailed).

SRG, Self-Regulation; NRG, Nonregulation; DGR, Dysregulation; ER, External Regulation; ENRG, External Nonregulation; EDRG, External Dysregulation Regulation.

### 3.3 Linear prediction relationships

#### 3.3.1 Prediction of regulation factors with psychological strengths

**Figure 1:**
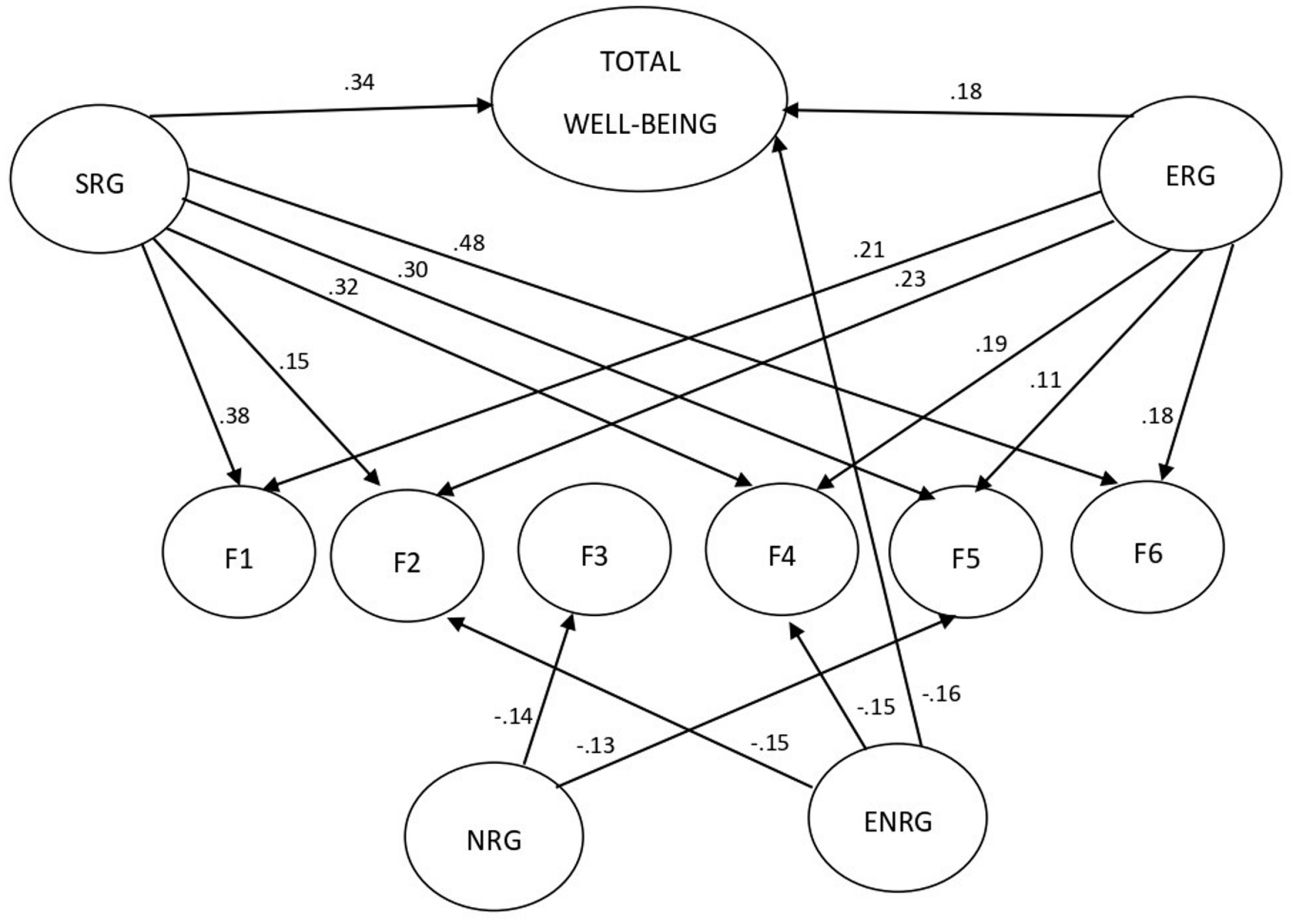
Effect of regulation levels (SRG, ERG, NRG, and ENRG) on psychological well-being: F1. Self-acceptance, F2. Positive relations with others, F3. Autonomy, F4. Environmental mastery, F5. Personal growth, F6. Purpose in life.

The combination of the six dimensions comprising the regulatory framework yielded a significant and positive prediction of total strengths [*F* (6,447) = 23.43, *p* < 0.0001; *R*^2^ = 24%]. The examination of each dimension individually revealed a statistically significant positive relationship between Self- Regulation (SRG; β = 0.36, *p* < 0.001) and External Regulation (ER; β = 0.26, *p* < 0.001) and total strength. A significant relationship was also observed between the levels of Self and External Regulation and each of the dimensions of strengths. Furthermore, the data showed a significant negative relationship between Self Dysregulation and the justice dimension (DRG; β = −0.13, *p* < 0.05) and, conversely, a positive relationship between Self Dysregulation and transcendence (DRG; β = 0.16, *p* < 0.01). In addition, a significantly positive relationship was observed between External Non-Regulation and the Temperance dimension (ENR; β = 0.13, *p* < 0.03) (Table 4; Figure 1).

**Table 4:** Predictive relationships of types of regulation with each of the dimensions of psychological strengths.

VD (Fortalezas)	F	gl	*R* ^2^	SRG	NRG	DRG	ERG	ENRG	EDRG
D1. Wisdom and knowledge	27.90	(6, 446)	0.27	0.48 (<0.001)**, IC [0.36, 0.52]	0.01 (0.897), IC [−0.08, 0.09]	0.08 (0.166), IC [−0.03, 0.15]	0.12 (0.011)*, IC [0.02, 0.14]	0.08 (0.142), IC [−0.02, 0.13]	−0.11 (0.066), IC [−0.14, 0.00]
D2. Courage	32.45	(6, 446)	0.30	0.46 (<0.001)**, IC [0.32, 0.47]	0.03 (0.518), IC [−0.05, 0.10]	0.10 (0.086), IC [−0.01, 0.15]	0.17 (<0.001)**, IC [0.05, 0.17]	0.01 (0.910), IC [−0.06, 0.07]	−0.07 (0.228), IC [−0.10, 0.03]
D3. Humanity	28.51	(6, 446)	0.28	0.38 (<0.001)**, IC [0.26, 0.41]	0.06 (0.251), IC [−0.03, 0.12]	−0.01 (0.923), IC [−0.09, 0.08]	0.25 (<0.001)**, IC [0.11, 0.23]	−0.04 (0.523), IC [−0.09, 0.05]	−0.04 (0.440), IC [−0.09, 0.04]
D4. Justice	23.69	(6, 446)	0.24	0.40 (<0.001)**, IC [0.27, 0.43]	0.04 (0.436), IC [−0.05, 0.11]	−0.13 (0.034)*, IC [−0.18, −0.01]	0.20 (<0.001)**, IC [0.07, 0.19]	0.07 (0.225), IC [−0.03, 0.12]	−0.06 (0.312), IC [−0.10, 0.03]
D5. Temperance	17.92	(6, 446)	0.19	0.36 (<0.001)**, IC [0.23, 0.39]	−0.01 (0.799), IC [−0.09, 0.07]	−0.09 (0.141), IC [−0.15, 0.02]	0.20 (<0.001)**, IC [0.07, 0.19]	0.13 (0.026)*, IC [0.01, 0.16]	−0.02 (0.803), IC [−0.08, 0.06]
D6. Trascendence	29.63	(6, 446)	0.29	0.43 (<0.001)**, IC [0.32, 0.47]	0.03 (0.534), IC [−0.06, 0.11]	0.16 (0.005)**, IC [0.04, 0.21]	0.17 (<0.001)**, IC [0.05, 0.17]	0.01 (0.899), IC [−0.07, 0.08]	−0.08 (0.169), IC [−0.12, 0.02]
Total strengths	23.43	(6, 446)	0.24	0.36 (<0.001)**, IC [0.20, 0.33]	−0.02 (0.723), IC [−0.08, 0.06]	0.00 (0.962), IC [−0.07, 0.07]	0.26 (<0.001)**, IC [0.09, 0.20]	0.14 (0.015)*, IC [0.01, 0.13]	−0.07 (0.227), IC [−0.09, 0.02]

**The correlation is significant at the 0.01 level (two-tailed).

*The correlation is significant at the 0.05 level (two-tailed).

SRG, Self-Regulation; NRG, Nonregulation; DGR, Dysregulation; ER, External Regulation; ENRG, External Nonregulation; EDRG, External Dysregulation Regulation.

The bold values indicate the statistically significant results in the analyses.

#### 3.3.2 Self and external regulation as predictors of psychological well-being

**Figure 2:**
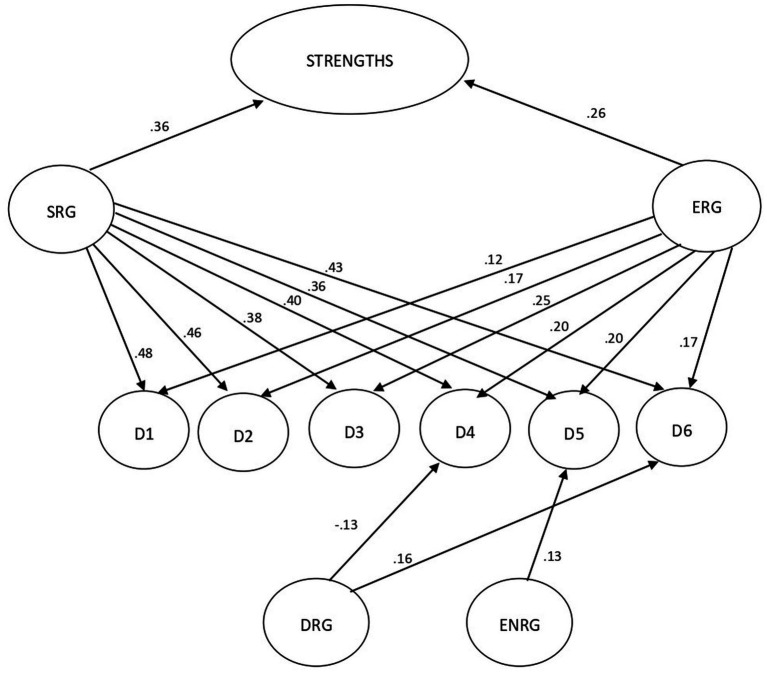
Effect of regulation levels (SRG, ERG, NRG, and ENRG) on psychological Strengths: D1. Wisdom and Knowledge, D2. Courage, D3. Humanity, D4. Justice, D5. Temperance, D6. Trascendence.

The predictive findings consistently demonstrated that the regulatory construct significantly and positively forecasted the total well-being score [*F* (6,447) = 30.07, *p* < 0.0001; *R*^2^ = 29%]. Notably, the total well-being score was significantly and positively predicted by the dimensions of Self- Regulation (SRG; β = 0.34, *p* < 0.001) and External Regulation (ERG; β = 0.18, *p* < 0.001). Additionally, the results reveal a significant negative prediction between External Nonregulation and total well-being (ENRG; β = −0.16; *p* < 0.007). Focusing on each dimension of well-being, all of the dimensions were positively related to the levels of Self and External Regulation (Table 5; Figure 2). On the other hand, a significantly negative relationship was shown between self-nonregulation and the dimensions of autonomy (NRG; β = −0.14; *p* < 0.01) and personal growth (NRG; β = 0.13; *p* < 0.01).

**Table 5:** Predictive relationships of types of regulation with each of the dimensions of the factors of psychological well-being.

Variable	F	gl	*R* ^2^	SRG	NRG	DRG	ERG	ENRG	EDRG
F1. Self-acceptance	31.24	(6, 446)	0.30	0.38 (<0.001)**, IC [0.44, 0.69]	−0.07 (0.145), IC [−0.23, 0.03]	0.09 (0.129), IC [−0.03, 0.24]	0.21 (<0.001)**, IC [0.13, 0.33]	−0.10 (0.076), IC [−0.22, 0.01]	0.03 (0.594), IC [−0.08, 0.14]
F2. Positive relations with others	17.42	(6, 446)	0.19	0.15 (0.001)**, IC [0.10, 0.41]	0.01 (0.835), IC [−0.14, 0.17]	−0.12 (0.066), IC [−0.32, 0.01]	0.23 (<0.001)**, IC [0.17, 0.40]	−0.15 (0.012)*, IC [−0.32, −0.04]	−0.04 (0.473), IC [−0.18, 0.09]
F3. Autonomy	5.67	(6, 446)	0.07	0.10 (0.058), IC [−0.00, 0.26]	−0.14 (0.014)*, IC [−0.30, −0.03]	0.01 (0.905), IC [−0.13, 0.15]	0.05 (0.362), IC [−0.05, 0.15]	−0.10 (0.102), IC [−0.22, 0.02]	0.00 (0.995), IC [−0.11, 0.11]
F4. Environmental mastery	23.36	(6, 446)	0.24	0.32 (<0.001)**, IC [0.30, 0.54]	−0.04 (0.418), IC [−0.17, 0.07]	−0.03 (0.651), IC [−0.16, 0.10]	0.19 (<0.001)**, IC [0.09, 0.28]	−0.15 (0.010)*, IC [−0.25, −0.03]	0.06 (0.292), IC [−0.05, 0.16]
F5. Personal growth	20.60	(6, 446)	0.22	0.30 (<0.001)**, IC [0.27, 0.51]	−0.13 (0.016)*, IC [−0.26, −0.03]	−0.09 (0.162), IC [−0.22, 0.04]	0.11 (0.019)*, IC [0.02, 0.20]	−0.08 (0.179), IC [−0.18, 0.03]	−0.01 (0.888), IC [−0.11, 0.09]
F6. Purpose in life	40.64	(6, 446)	0.35	0.48 (<0.001)**, IC [0.59, 0.84]	−0.02 (0.629), IC [−0.16, 0.10]	0.03 (0.584), IC [−0.10, 0.17]	0.18 (<0.001)**, IC [0.11, 0.30]	−0.07 (0.166), IC [−0.19, 0.03]	0.03 (0.522), IC [−0.07, 0.14]
Total well-being	30.07	(6, 446)	0.29	0.34 (<0.001)**, IC [0.27, 0.46]	−0.09 (0.074), IC [−0.18, 0.01]	−0.00 (0.970), IC [−0.10, 0.10]	0.18 (<0.001)**, IC [0.08, 0.22]	−0.16 (0.004)**, IC [−0.21, −0.04]	0.01 (0.907), IC [−0.08, 0.09]

**The correlation is significant at the 0.01 level (two-tailed).

*The correlation is significant at the 0.05 level (two-tailed).

SRG, Self-Regulation; NRG, Nonregulation; DGR, Dysregulation; ER, External Regulation; ENRG, External Nonregulation; EDRG, External Dysregulation Regulation.

The bold values indicate the statistically significant results in the analyses.

#### 3.3.3 Prediction of character strengths regarding Total psychological well-being

The dimensions of character strengths significantly predicted total well-being [*F* (6,447) = 47.07; *p* < 0.001; *R*^2^ = 39%], with particularly strong predictive power from the dimensions humanity [β = 0.39, *p* < 0.001] and courage [β = 0.33, *p* < 0.01] (Table 6).

**Table 6:** Predictive relationships of dimensions of psychological strengths and the psychological well-being.

VD	F	gl	*R* ^2^	D1. Wisdom and knowledge	D2. Courage	D3. Humanity	D4. Justice	D5. Temperance	D6. Trascendence
Total well-being	47.07	(6, 446)	0.39	−0.02 (0.767), IC [−0.16, 0.12]	0.33 (<0.001)**, IC [0.25, 0.58]	0.39 (<0.001)**, IC [0.34, 0.62]	−0.09 (0.119), IC [−0.25, 0.03]	0.02 (0.717), IC [−0.10, 0.14]	0.05 (0.451), IC [−0.09, 0.19]

**The correlation is significant at the 0.01 level (two-tailed).

*The correlation is significant at the 0.05 level (two-tailed).

SRG, Self-Regulation; NRG, Nonregulation; DGR, Dysregulation; ER, External Regulation; ENRG, External Nonregulation; EDRG, External Dysregulation Regulation.

The bold values indicate the statistically significant results in the analyses.

### 3.4 Structural prediction

A structural prediction model was tested involving six predictive factors—three for Self-Regulation (SRG, NRG, and DRG) and three for External Regulation (ERG, ENRG, and EDRG)—in relation to the dimensions of character strengths (as a mediator variable) and psychological well-being (Table 7). All fit indices of the incremental model were above the threshold of 0.90 (Bentler, 1990). The CFI was 0.928, which was also satisfactory. The RMSEA was 0.08, which lies within the range of acceptable values (Ho, 2006).

**Table 7:** Models of structural linear.

Factors	*X*^2^ (GL = CMIN/DF *p <*.)	NFI	RFI	IFI	TLI	CFI	RMSEA	H (*p* < 0.05)	H (*p* < 0.01)
F	Ò911.322(1,071) = 2718 (*p* <.001)	0.80	0.78	0.86	0.85	0.86	0.06	179	184

## 4 Discussion

The present study examined a linear predictive and structural mediation model aimed at explaining the relationships among Self and External Behavioral Regulation, psychological strengths, and psychological well-being in university students. Previous research has consistently reported positive associations between psychological strengths and well-being (Azañedo et al., 2021; Wood et al., 2011; Zhang and Chen, 2018). However, these studies have also emphasized the need to further explore the psychological and behavioral mechanisms underlying such associations. This approach will help to better understand such relationships and design more effective treatment plans (Azañedo et al., 2021; Zhang and Chen, 2018). In response to this gap, the present research investigated whether regulatory behavior processes contribute to explaining the relationship between strengths and well-being, and whether psychological strengths operate as mediating variables in this association.

### 4.1 Self–external regulation and psychological strengths

Consistent with the first hypothesis, the results revealed significant positive associations and predictive relationships between Self and External Regulation and psychological strengths, both at the global level and across specific dimensions. In contrast, Nonregulation and Dysregulation factors showed negative associations, although only Self and External Regulation emerged as robust predictors in the linear models. Notably, approximately 37% of the variance in psychological strengths was accounted for by these regulatory factors.

These findings suggest that psychological strengths are more strongly associated with adaptive regulatory patterns, particularly when personal regulatory capacities are supported by a facilitative context. From a theoretical perspective, this pattern aligns with the Self- vs. External-Regulation of Behavior Theory (de la Fuente et al., 2021, 2022a, 2022b), which emphasizes that behavioral regulation should be understood as the result of the interaction between individual competencies and contextual regulatory conditions. Rather than emerging solely from internal traits, psychological strengths appear to be fostered within regulatory environments that encourage goal-directed behavior, monitoring, and adjustment.

While earlier studies have reported associations between self-regulation and strengths (Artuch-Garde et al., 2017; Nota et al., 2004) or between perseverance and effort (Mrazek et al., 2018), these investigations primarily focused on individual-level variables. The present study extends this literature by showing that external regulatory conditions also play a relevant predictive role, thereby offering a more integrative account of how psychological strengths may develop. Importantly, these findings do not suggest that regulation causes strengths. Rather, they indicate that regulatory tendencies—both personal and contextual—are systematically associated with higher levels of strengths. This distinction is crucial because regulatory contexts may provide opportunities for the expression and reinforcement of strengths rather than producing them directly.

The analysis of specific strengths offers additional insights into the nuanced role of regulatory behavior. For example, the strength of justice showed positive associations with Self and External Regulation and negative associations with Nonregulation and Dysregulation. Previous research has suggested that justice-oriented behavior requires sustained cognitive effort and self-monitoring, particularly in contexts involving fatigue or moral conflict (Whiteside and Barclay, 2018). From this perspective, the present findings suggest that justice may be more readily expressed in individuals who possess sufficient regulatory resources and operate within contexts that reinforce fairness norms. This interpretation is consistent with neurocognitive accounts highlighting the regulatory demands involved in moral decision-making and norm enforcement (Buckholtz and Marois, 2012).

Similarly, integrity, located within the courage virtue, emerged as one of the most consistently associated strengths, showing positive relationships with Self and External Regulation and negative associations with dysregulatory patterns. Prior studies have linked integrity to self-regulatory processes supporting healthy and value-consistent behavior (Stone and Focella, 2011). In the present study, integrity also functioned as a mediating strength between regulatory behavior and well-being, suggesting that regulatory tendencies may be associated with well-being partly through the facilitation of value-driven behavior. Therefore, integrity proved to be the most consistent strength within the Self- vs. External Regulation of Behavior Theory (de la Fuente et al., 2021; de la Fuente et al., 2022a, 2022b). Again, this mediation should be understood as a predictive pathway rather than a causal mechanism.

An especially novel contribution of this study concerns the positive associations observed between certain transcendence-related strengths (e.g., spirituality, hope, humor) and Dysregulation factors. Although counterintuitive, this pattern suggests that limited or context-dependent dysregulation may coexist with, or even accompany, specific strengths that enable individuals to persist in challenging or uncertain situations. For example, transcendence showed positive associations with Self and External Dysregulation. While empirical evidence on this relationship remains scarce, these findings may be interpreted within frameworks that conceptualize transcendence as a resource for meaning-making under adversity (Kim et al., 2024). In such contexts, rigid adherence to regulatory norms may be temporarily relaxed, allowing individuals to persist based on faith, hope, or existential commitment rather than instrumental cost–benefit evaluations. This interpretation does not imply that dysregulation is beneficial per se, but rather that certain strengths may operate differently under non-normative or stressful conditions. The roles of spirituality and hope further illustrate this complexity. Prior research has yielded mixed findings regarding their associations with well-being (Eichhorn, 2012; Leondari and Gialamas, 2009), likely due to conceptual, cultural, and measurement differences (González-Rivera et al., 2017). The present findings align with studies reporting positive associations between spirituality, hope, well-being, and Self-Regulation (Abente et al., 2022; Villani et al., 2019), while also indicating that these strengths may be activated under dysregulatory conditions. This interpretation is consistent with growing evidence supporting the effectiveness of interventions that incorporate spiritual or existential components (Darvishi et al., 2020; Koenig et al., 2016). Similarly, humor displayed positive associations with both Regulation and Dysregulation, suggesting the coexistence of adaptive and maladaptive humor strategies. Adaptive humor may expand coping options and cognitive flexibility, whereas maladaptive humor may trivialize adversity and reduce perceived agency. This duality aligns with prior work highlighting differential emotional outcomes depending on humor style (Mathews, 2016).

Regarding psychological well-being, the results supported a positive predictive association with Self and External Regulation. Previous studies have linked individual self-regulatory processes to well-being (Tangney et al., 2004; Nyklíček et al., 2011); however, these investigations typically focused on intrapersonal variables. The present study extends this literature by demonstrating that behavioral regulation related to well-being operates at both personal and contextual levels. Nevertheless, the stronger predictive weight of individual regulation relative to external regulation aligns with prior findings suggesting that young adults’ well-being is more closely linked to personal agency than to contextual control (Hofer et al., 2011). This pattern underscores the importance of considering developmental context when interpreting regulatory influences.

### 4.2 Mediating role of psychological strengths

In line with the second hypothesis, psychological strengths emerged as mediators in the relationship between Self–External Regulation and well-being. This finding integrates prior evidence indicating that self-regulatory processes are associated with well-being (van Genugten et al., 2017), that self-regulation is linked to higher strengths (Artuch-Garde et al., 2017), and that strengths contribute to well-being (Park and Peterson, 2008). From a theoretical standpoint, psychological strengths may function as mechanisms that translate regulatory tendencies into well-being-related experiences, by facilitating effective coping, meaning-making, and engagement. This mediating role reflects predictive associations rather than causal pathways, for example, individuals with strong self-regulation skills are better equipped to leverage their strengths, allowing them to face challenges and experience well-being. Therefore, developing and implementing psychological strengths can be a useful strategy for enhancing the effects generated of behavioral regulation and increasing the individual’s well-being.

However, our investigation presents various limitations that warrant consideration. First, the explorations were conducted through self-report instruments; the data provide information on the participants’ perceptions of their own self and external regulation processes, which can lead to biased data. Second, the results are based on predictive analyses, which means that the inferences from this study should be adjusted to their predictive nature and should not be interpreted as causal relationships between the analyzed variables. Regarding the scenario under study, the data referred to a higher-education setting, which means that inferences can be made only with respect to such a context. Moreover, the predominance of women in the sample may have influenced the study outcomes. The overrepresentation of female participants could have biased the findings toward patterns more characteristic of women. This issue is particularly relevant given prior evidence of gender differences in character strengths among young populations (Furnham and Lester, 2012). Thus, the results may not be generalizable to the broader undergraduate population. Additionally, the convenience sampling method captures the characteristics of the accessible population rather than offering a fully representative picture of Spanish undergraduates overall. These considerations highlight the need for caution when generalizing the findings and encourage the development of studies that intend to identify the potential fit of the Self- vs. External Regulation Behavior Model in other populations, particularly in communities or clinical settings.

## 5 Conclusion

The findings of this research highlight the significance of both Self and External Behavioral Regulation as key mechanisms in fostering psychological strengths that enhance the likelihood of well-being among young university students. Moreover, the data support the usefulness of the Self vs. External Regulation Behavior Theory (de la Fuente et al., 2021; de la Fuente et al., 2022a, 2022b) in providing a deeper understanding of individuals’ experiences and their interactions with potentially challenging contexts.

## 6 Practical implications

The data from this research provide evidence of how the Self vs. External Regulation Behavior Theory (de la Fuente et al., 2021; de la Fuente et al., 2022a, 2022b) is cross-sectional and applicable in other settings beyond education, specifically in the field of clinical and health Psychology. Studies such as the present one might be useful for contextual therapies by highlighting how important the relationship between the individual and their context.

Numerous studies have documented the advantages of therapies such as the acceptance and commitment therapy (ACT) in helping individuals cope with emotional problems and improving the well-being of young people; these therapies consider both their personal resources and their interactions with the context that shapes them (Levin et al., 2014; Räsänen et al., 2016). Therefore, it is considered relevant to develop studies that link the evaluation and intervention of Self and External Regulatory processes within the development of third-generation therapies, as these studies provide elements that promote well-being and health in individuals. Along these lines, different interventions integrate spiritual aspects into their design, such as Religiously Integrated Cognitive–Behavioral Therapy (RCBT), which yields positive results for depressive and anxiety symptoms (Koenig et al., 2016; Paukert et al., 2009) and suicidal thoughts (Ramos et al., 2018), as well as for the treatment of addictions (Hodge and Lietz, 2014). Furthermore, Spiritual Therapy has yielded positive results for the well-being of subjects (Darvishi et al., 2020), which supports the idea of considering transcendent dimensions not previously addressed. Additionally, current review studies have confirmed the effectiveness of psychological treatments that include humor in their interventions (Sarink and García-Montes, 2023; Stiwi and Rosendahl, 2022).

Furthermore, clinical and health Psychology might find of interest the implementation of studies that include a sample of clinical subjects who present psychological distress or mental pathologies to analyse the applicability of the model in those contexts. Investigating the impact of Regulation in various domains associated with clinical and health psychology will contribute to obtaining more precise and accurate insights into the influence of this construct across diverse population groups, encompassing both general and clinical contexts. This approach facilitates the development of specific tools and treatment plans tailored to each unique scenario.

## Data Availability

The datasets presented in this article are not readily available due to privacy and ethical restrictions. Requests to access the datasets should be directed to eoswaldo@unav.es.
