# Supplementary material for: The role of psychological strengths in the relationship between self- and external- regulation of behavior and psychological well-being of university students
Source: Front Psychol. 2026 Apr 2;17:1641359. doi: 10.3389/fpsyg.2026.1641359 (PMC13083076; doi:10.3389/fpsyg.2026.1641359)
Supplement: Supplementary file 1 [file Table_1.docx]

**Table 1**

*Descriptive statistics. Self-External Regulation values (n=453)*

| Self-External Regulation | | | Psychological Strengths | | | Psychological Well-being | | |
| --- | --- | --- | --- | --- | --- | --- | --- | --- |
| Dimension | Mean (M) | SD | Dimension | Mean (M) | SD | Dimension | Mean (M) | SD |
| SRG | 4.01 | .31 | D1. Wisdom and Knowledge | 3.65 | .28 | F1. Self-acceptance | 4.54 | .46 |
| NRG | 2.59 | .35 | *F1. Curiosity* | 3.48 | .34 | F2. Positive relations with others | 4.46 | .52 |
| DRG | 2.57 | .38 | *F2. Love of Learning* | 3.32 | .32 | F3. Autonomy | 4.03 | .41 |
| ERG | 3.81 | .41 | *F3. Open-mindedness* | 3.74 | .34 | F4. Environmental mastery | 4.17 | .41 |
| ENRG | 2.43 | .43 | *F4. Creativity* | 3.73 | .34 | F5. Personal growth | 4.89 | .39 |
| EDRG | 2.47 | .45 | *F5. Perspective* | 3.98 | .29 | F6. Purpose in life | 4.51 | .46 |
|  |  |  | D2. Courage | 3.81 | .26 |  |  |  |
|  |  |  | *F6. Bravery* | 3.58 | .33 |  |  |  |
|  |  |  | *F7. Persistence* | 3.67 | .38 |  |  |  |
|  |  |  | *F8. Integrity* | 4.25 | .28 |  |  |  |
|  |  |  | *F9. Vitality* | 3.75 | .35 |  |  |  |
|  |  |  | D3. Humanity | 3.96 | .27 |  |  |  |
|  |  |  | *F10. Love* | 3.92 | .39 |  |  |  |
|  |  |  | *F11. Kindness* | 4.18 | .27 |  |  |  |
|  |  |  | *F12. Social intelligence* | 3.82 | .30 |  |  |  |
|  |  |  | D4. Justice | 4.00 | .27 |  |  |  |
|  |  |  | *F13. Citizenship* | 4.11 | .30 |  |  |  |
|  |  |  | *F14. Fairness* | 3.92 | .27 |  |  |  |
|  |  |  | *F15. Leadership* | 3.99 | .30 |  |  |  |
|  |  |  | D5. Temperance | 3.56 | .27 |  |  |  |
|  |  |  | *F16. Forgiveness and Mercy* | 3.40 | .43 |  |  |  |
|  |  |  | *F17. Humility and Modesty* | 3.57 | .33 |  |  |  |
|  |  |  | *F18. Prudence* | 3.84 | .34 |  |  |  |
|  |  |  | *F19. Self-regulation* | 3.42 | .35 |  |  |  |
|  |  |  | D6. Trascendence | 3.51 | .28 |  |  |  |
|  |  |  | *F20. Appreciation of Beauty and Excellence* | 3.61 | .34 |  |  |  |
|  |  |  | *F21. Gratitude* | 3.75 | .30 |  |  |  |
|  |  |  | *F22. Hope* | 3.55 | .40 |  |  |  |
|  |  |  | *F23. Humor* | 3.86 | .37 |  |  |  |
|  |  |  | *F24. Spirituality* | 2.77 | .48 |  |  |  |
| Total |  |  | Total Dimensions  Total Factor | 3.76  3.74 | .23  .02 | Total Well-Being | 4.44 | .33 |

Note. SRG= Self-Regulation; NRG=Nonregulation; DGR= Dysregulation: ER= External Regulation; ENRG=External Nonregulation; EDRG= External Dysregulation Regulation

**Table 2**

*Association relationships. Self and External Regulation and Psychological Strengths Factors (n=453)*

| Factor | SRG | NRG | DRG | ERG | ENRG | EDRG |
| --- | --- | --- | --- | --- | --- | --- |
| F1. Curiosity | **.47 (< .001)**** | −.03 (.48) | .09 (.05) | **.30 (< .001)**** | −.03 (.48) | −.03 (.50) |
| F2. Love of Learning | **.44 (< .001)**** | **−.11 (.02)*** | .04 (.35) | **.24 (< .001)**** | **−.11 (.02)*** | **−.11 (.02)*** |
| F3. Open-mindedness | **.43 (< .001)**** | −.05 (.29) | .03 (.55) | **.21 (< .001)**** | −.05 (.29) | −.05 (.29) |
| F4. Creativity | **.45 (< .001)**** | −.01 (.82) | **.11 (.02)*** | **.19 (< .001)**** | −.01 (.82) | −.01 (.82) |
| F5. Perspective | **.55 (< .001)**** | **−.12 (.01)*** | −.03 (.59) | **.33 (< .001)**** | **−.12 (.01)*** | **−.12 (.01)*** |
| F6. Bravery | **.42 (< .001)**** | −.02 (.63) | **.12 (.01)*** | **.20 (< .001)**** | −.02 (.63) | −.02 (.63) |
| F7. Persistence | **.44 (< .001)**** | −.02 (.69) | .05 (.31) | **.34 (< .001)**** | −.02 (.69) | −.02 (.69) |
| F8. Integrity | **.38 (< .001)**** | **−.14 (.00)**** | **−.13 (.01)*** | **.28 (< .001)**** | **−.14 (.00)**** | **−.14 (.00)**** |
| F9. Vitality | **.51 (< .001)**** | −.06 (.20) | .04 (.37) | **.41 (< .001)**** | −.06 (.20) | −.06 (.20) |
| F10. Love | **.38 (< .001)**** | −.09 (.05) | −.04 (.40) | **.38 (< .001)**** | −.09 (.05) | −.09 (.05) |
| F11. Kindness | **.42 (< .001)**** | **−.13 (.01)*** | **−.10 (.03)*** | **.38 (< .001)**** | **−.13 (.01)*** | **−.13 (.01)*** |
| F12. Social intelligence | **.44 (< .001)**** | −.02 (.68) | **.10 (.03)*** | **.33 (< .001)**** | −.02 (.68) | −.02 (.68) |
| F13. Citizenship | **.32 (< .001)**** | **−.12 (.01)*** | **−.10 (.03)*** | **.28 (< .001)**** | **−.12 (.01)*** | **−.12 (.01)*** |
| F14. Fairness | **.31 (< .001)**** | **−.14 (.00)**** | **−.13 (.01)*** | **.29 (< .001)**** | **−.14 (.00)**** | **−.14 (.00)**** |
| F15. Leadership | **.39 (< .001)**** | **−.16 (.00)**** | **−.11 (.02)*** | **.34 (< .001)**** | **−.16 (.00)**** | **−.16 (.00)**** |
| F16. Forgiveness and Mercy | **.29 (< .001)**** | −.09 (.05) | −.04 (.40) | **.25 (< .001)**** | −.09 (.05) | −.09 (.05) |
| F17. Humility and Modesty | **.21 (< .001)**** | −.08 (.08) | −.06 (.18) | **.19 (< .001)**** | −.08 (.08) | −.08 (.08) |
| F18. Prudence | **.27 (< .001)**** | **−.10 (.03)*** | −.09 (.05) | **.22 (< .001)**** | **−.10 (.03)*** | **−.10 (.03)*** |
| F19. Self-regulation | **.46 (< .001)**** | **−.17 (< .001)**** | **−.19 (< .001)**** | **.36 (< .001)**** | **−.17 (< .001)**** | **−.17 (< .001)**** |
| F20. Appreciation of Beauty and Excellence | **.53 (< .001)**** | **−.12 (.01)*** | −.03 (.59) | **.33 (< .001)**** | **−.12 (.01)*** | **−.12 (.01)*** |
| F21. Gratitude | **.55 (< .001)**** | **−.11 (.02)*** | **.11 (.02)*** | **.33 (< .001)**** | **−.11 (.02)*** | **−.11 (.02)*** |
| F22. Hope | **.57 (< .001)**** | −.09 (.05) | **.10 (.03)*** | **.36 (< .001)**** | −.09 (.05) | −.09 (.05) |
| F23. Humor | **.49 (< .001)**** | −.07 (.14) | .09 (.05) | **.31 (< .001)**** | −.07 (.14) | −.07 (.14) |
| F24. Spirituality | **.24 (< .001)**** | **.20 (< .001)**** | **.24 (< .001)**** | **.17 (< .001)**** | **.14 (.00)**** | **.21 (< .001)**** |

**. The correlation is significant at the 0.01 level (two-tailed).

*. The correlation is significant at the 0.05 level (two-tailed).

Note. SRG= Self-Regulation; NRG=Nonregulation; DGR= Dysregulation: ER= External Regulation; ENRG=External Nonregulation; EDRG= External Dysregulation Regulation
